# Association between diet and quality of life among healthcare professionals in King Saud University Medical City

**DOI:** 10.3389/fpubh.2025.1595412

**Published:** 2025-08-01

**Authors:** Waad Alfawaz, Reem S. Albassam, Noura Almuharib, Shuruq Alghafis, Walaa Mahfouz

**Affiliations:** ^1^Department of Community Health Sciences, College of Applied Medical Sciences, King Saud University, Riyadh, Saudi Arabia; ^2^Department of Scientific Research, King Saud University, Riyadh, Saudi Arabia

**Keywords:** diet, quality of life, healthcare professionals, WHOQOL, RDQS

## Abstract

**Background:**

A balanced diet and active lifestyle are key factors influencing health and quality of life (QoL). This study examined the relationships between eating patterns, physical activity, and QoL among Arab healthcare professionals.

**Methodology:**

A cross-sectional study included 353 healthcare workers in Riyadh, Saudi Arabia. Data were collected through face-to-face interviews, where participants were briefed on the study objectives and provided voluntary informed consent. The WHOQOL-BREF tool was used to evaluate QoL, while the Rapid Diet Quality Screener assessed dietary habits. The Global Physical Activity Questionnaire was used to measure physical activity. Statistical analysis included independent samples t-tests, one-way ANOVA, and Pearson correlation.

**Results:**

Among participants, 47.6% had a normal weight, 44.7% were overweight or obese, and 7.6% were underweight. While those with normal weight had the highest QoL scores across all domains, differences across BMI categories were not statistically significant. Regular physical activity was linked to high QoL, particularly in social relationships (*p* = 0.037). Participants who exercised regularly also reported healthier eating habits (*p* < 0.001). Weak but significant positive correlations were found between diet quality and QoL, particularly physical health (*r* = 0.219, *p* < 0.001) and psychological health (*r* = 0.184, *p* = 0.002). No significant diet quality differences were found between genders (*p* = 0.677).

**Conclusion:**

Healthcare professionals who exercise regularly exhibit a better QoL, especially in social relationships, and maintain healthier eating habits. While normal BMI was associated with improved QoL, differences across BMI categories were not statistically significant. These findings highlight the need for interventions promoting physical activity and healthy eating to enhance QoL in healthcare workers.

## 1 Introduction

A balanced diet has long been recognized as a key factor in maintaining good health and an overall quality of life (QoL) (1). Physical inactivity and unhealthy eating behaviors contribute significantly to economic burdens, including lost productivity at work. When healthcare workers are affected, it can indirectly impact the efficiency of the healthcare sector (2).

Several studies have assessed the healthy lifestyle and cardiometabolic risk among healthcare staff and found that the majority adhere to unhealthy diets and engage in low levels of physical activity (2, 3). A study from United Kingdom evaluated the incidence of cardiovascular risk factors and adherence to dietary and physical activity recommendations among National Health Service staff (2). It reported that half of the participants were overweight or obese, with low compliance with dietary and physical activity guidelines (2). Another study from South Africa indicated that nurses are at a higher risk of non-communicable diseases, with a high prevalence of poor dietary habits, obesity, and insufficient physical activity (3). Sharma et al. (4) conducted a study in India examining the prevalence of risk factors for lifestyle diseases among healthcare professionals. The study reported that the prevalence of diabetes and hypertension in this population was 5 and 10%, respectively, both of which are associated with lifestyle and dietary factors (4). A study conducted in Saudi Arabia revealed that a significant proportion of healthcare professionals across various specialties and practice settings exhibit unhealthy dietary behaviors both in the workplace and in their personal lives. These behaviors are characterized by excessive consumption of sweets and coffee, along with frequent episodes of binge eating. The study identified key contributing factors, including demanding workloads, insufficient break times, and limited availability of nutritious food options within hospital environments (5). A cohort study comprised 11,128 individuals from a cohort known as “Seguimiento Universidad de Navarra” (SUN). Dietary practices were evaluated using a validated food frequency questionnaire. QoL was measured using the validated Spanish version of the SF-36 Health Survey. This study aimed to determine the relationship between dietary habits and emotional and physical quality of life, following a 4-year follow-up period. The researchers found that after 4 years of follow-up, baseline adherence to a Mediterranean dietary pattern was directly associated with higher QoL scores in the SUN Project, while baseline adherence to a Western dietary pattern was inversely associated with self-perceived QoL (6). Phiri et al. (3) observed that nurses who exercised regularly experienced lower stress levels, better physical health, and improved social interactions, all contributing to enhanced QoL. A recent study assessing the QoL among healthcare workers in the Arab world found that the majority reported suboptimal QoL scores across various domains, including physical, psychological, social, and environmental aspects. However, general QoL scores remained relatively satisfactory (7). A study in Saudi Arabia also reported poor QoL among healthcare professionals working in four primary cares centers (8). Doctors frequently neglect their physical and mental health and exhibit higher levels of anxiety, burnout, depression, and substance misuse than other occupational groups (9, 10).

Our research is pioneering in its examination of the interconnections between dietary habits, physical activity, and QoL among healthcare professionals in Saudi Arabia. This study provides region-specific insights and adds to the sparse body of research on Middle Eastern healthcare populations, where previously these factors were often investigated in isolation. By integrating nutrition, physical activity, and QoL within a single healthcare cohort, providing a holistic approach unlike to the previous regional research that often examines these factors separately. Analyzing these interconnected lifestyle dimensions offers a more comprehensive perspective of well-being. Workplace interventions to improve nutrition and quality of life, including work-related outcomes, are complex and challenging to implement owing to a lack of understanding (1). The association between diet and QoL among medical staff is a crucial area of study since healthcare professionals’ well-being directly influences their ability to provide effective patient care and educate medical students (2). However, the specific relationship between dietary choices, lifestyle factors and QoL among medical staff remains unclear. Understanding this relationship is essential for identifying potential interventions and strategies to enhance the well-being and work-life balance of medical professionals. Based on the findings of our study, targeted interventions, such as providing healthy food options in the workplace, promoting healthy eating habits, and implementing stress management programs, can be developed to improve dietary habits and QoL among medical staff. These efforts are expected to improve patient outcomes and increase the effectiveness of health care systems. The current study aimed to determine the association between diet quality and QoL among medical staff, taking their lifestyle into account.

## 2 Materials and methods

### 2.1 Study design

This cross-sectional study was conducted at the King Saud University Medical City (KSUMC), Riyadh, Saudi Arabia. A total of 353 healthcare professionals were included in the study. The sample size was determined using the Epi calculator and Raosoft software (11) with a 95% confidence level, resulting in a sample of 353 participants. Data were collected through face-to-face interviews, which provided participants with information on the study objectives and ensured their voluntary consent to participate. Ethical approval was obtained from the Institutional Review Board (IRB) of King Saud University (KSU-HE-24-091) to ensure adherence to ethical standards and safeguard the rights and well-being of the participants.

### 2.2 Assessment tools

The World Health Organization QoL Instrument (WHOQOL-BREF) was used to assess the QoL of study participants. The WHOQOL-BREF is a self-administered questionnaire comprising 26 items measuring four keys QoL domains defined by the World Health Organization: physical health, psychological health, social relationships, and environment. The scores were transformed into a linear scale from 0 to 100, where 0 represented the lowest QoL, and 100 represented the highest (12). The first two items independently assessed overall perceptions of QoL and general health. Each question is rated on a 5-point Likert scale, ranging from 1 (very poor/very dissatisfied/none/never) to 5 (very good/very satisfied/extremely/always), with scores across the four domains summed and positively scaled, indicating higher QoL with higher scores (13). Numerous studies have validated the reliability of the WHOQOL-BREF as a QoL assessment tool (14, 15). The Arabic version has also been tested and shown to be valid and highly reliable among Arabic-speaking populations (Cronbach’s alpha > 0.867) (12).

The Rapid Diet Quality Screener (RDQS) developed by Kotecki et al. (16) was used to evaluate diet quality. This tool begins with the question, “In a typical week, how often do you:” and is followed by 12 questions regarding the frequency of consumption of certain foods and beverages associated with either reducing or increasing chronic disease risk. Six questions addressed high-quality foods (e.g., minimally processed vegetables, fruits, whole grains, protein-rich foods, and healthy fats), whereas the remaining six assessed low-quality foods (e.g., processed items, those high in added sugars, saturated and trans fats, and sodium, such as desserts, sugary drinks, red meat, and alcohol). For each item, responses are categorized into three frequency columns: “never/rarely,” “sometimes,” and “often/always.” This three-level frequency system minimizes recall and calculation errors. Responses were scored by dietary healthfulness, with the healthiest behavior scored as 2, a moderately healthful behavior as 1, and the least healthful behavior as 0. The total score represents the overall diet quality, ranging from 0 to 24. Higher scores reflect healthier dietary habits, whereas lower scores indicate areas requiring improvement. The score is then categorized into five diet quality levels for easy interpretation, each represented by a specific color: 21–24 (excellent, green), 17–20 (very good, light green), 14–16 (good, yellow), 7–13 (fair, orange), and 0–6 (poor, red). Effective screening tools such as the RDQS are designed not to quantify exact food and nutrient intake but rather to classify diets as relatively high or low in specific dietary qualities (16).

The Global Physical Activity Questionnaire (GPAQ), developed by the World Health Organization, was used to assess participants’ physical activity levels. GPAQ evaluates physical activity through a series of structured questions organized into three domains: occupational (work-related), transportation (such as walking and cycling), and recreational (leisure-time activities). Each domain assesses the frequency of these activities during a typical week, as well as the duration spent on each activity per day. Activity intensity is classified as either moderate or vigorous, with assigned Metabolic Equivalent Task (MET) values of 4.0 for moderate activities and 8.0 for vigorous activities. The total physical activity for each participant was calculated by multiplying these MET values by the reported duration of each activity (minutes per week), providing a continuous indicator of weekly energy expenditure expressed in MET-minutes per week. Then, categorical indicators of physical activity (insufficiently active, sufficiently active) were established following WHO recommendations. Participants were classified as sufficiently active if they engaged in at least 150 min of moderate-intensity physical activity, 75 min of vigorous-intensity physical activity, or an equivalent combination achieving at least 600 MET-minutes per week (17).

### 2.3 Statistical analyses

The data were preprocessed prior to analysis, including data cleaning; the dataset was examined for missing values, outliers, and consistency, and only complete cases were included in the final analysis. Descriptive statistics were employed to summarize the demographic variables, including sex, BMI categories, income levels, living area, walking activity, and physical activity. Independent samples t-tests were conducted to evaluate differences in mean scores by sex and physical activity status, while one-way ANOVA was used to compare mean differences across BMI categories. Additionally, the Pearson correlation coefficient was calculated to determine the strength and direction of the relationships between QoL domains and diet quality. Furthermore, multiple linear regression analysis was conducted to identify significant predictors of each QoL domain, using diet quality, physical activity levels, and relevant demographic variables as independent factors. Statistical significance was set at a *p*-value of <0.05. All data analyses were performed using IBM SPSS Statistics for Windows, Version 28.0 (IBM Corp., Armonk, NY, United States).

## 3 Results

### 3.1 General characteristics of the study sample

The study participants consist of 353 healthcare professionals employed at King Saud University’s Medical City. Among the participants, 56.9% were male and 43.1% were female. Nearly half (47.6%) of the respondents maintained a normal weight (BMI 18.5–24.9), while over 44% were overweight or obese.

The majority (52.1%) of participants earned ≤10,000 Saudi Riyals (SR) monthly, with 33.2% earned 10,000–20,000 SR, and 14.7% earned >20,000 SR. Most resided in Central (37.4%) and North Riyadh (30.3%).

Regarding walking activity, 13.9% did not walk in the past week. Among walkers, the most common frequency was 5 days (19%). Overall, 65.2% sufficiently active, whereas 34.8% insufficiently active. Among those participants, 15.6% exercised for 5 days a week, 14.4% for 3 days, and only 3.4% exercised daily (Table 1).

**Table 1:** Socio-demographic characteristics of the population (*n* = 353).

Items	*N*	%
Gender		
Male	201	56.9%
Female	152	43.1%
BMI		
Underweight: BMI less than 18.5	27	7.6%
Normal weight: BMI 18.5 to 24.9	168	47.6%
Overweight: BMI 25.0 to 29.9	109	30.9%
Obesity: BMI 30.0 and above	49	13.8%
Income		
10,000 SR or less	184	52.1%
10,000–15,000 SR	68	19.3%
15,000–20,000 SR	49	13.9%
More than 20,000 SR	52	14.7%
Living area		
Central Riyadh	132	37.4%
East of Riyadh	55	15.6%
North of Riyadh	107	30.3%
South of Riyadh	10	2.8%
West of Riyadh	37	10.5%
Outside Riyadh	12	3.4%
Walking activity (day’s walking > 30 min last week)		
No walking	49	13.9%
1 day	26	7.4%
2 days	41	11.6%
3 days	64	18.1%
4 days	36	10.2%
5 days	67	19.0%
6 days	21	5.9%
Daily 7 days	49	13.9%
Days of physical activity during the past week		
1 day	24	7.1%
2 days	38	10.8%
3 days	51	14.4%
4 days	40	11.3%
5 days	55	15.6%
6 days	10	2.8%
Daily 7 days	12	3.4%
Physical activity		
Insufficiently active	123	34.8%
Sufficiently active	230	65.2%

SR, Saudi Riyal; BMI, Body Mass Index. *p< 0.01.

### 3.2 Quality of life and RDQS according to impact factors

Table 2 illustrates the influence of gender, physical activity, and BMI on QoL domains and diet quality. The gender-based analysis showed that males scored slightly higher across all QoL domains (physical health, psychological health, social relations, and environment), with a significant advantage in social relations (68.0 ± 22.7 vs. 63.6 ± 18.1, *p* = 0.049). Diet quality did not differ significantly between sexes (*p* = 0.677).

**Table 2:** QoL and RDQS according to impact factors (*n* = 353).

Items	Quality of life (QoL)								RDQS	
	Physical health		Psychological health		Social relations		Environment			
	Mean ± SD	*P*-value	Mean ± SD	*P*-value	Mean ± SD	*P*-value	Mean ± SD	*P*-value	Mean ± SD	*P*-value
Gender										
Male (*n* = 201)	68.1 ± 16	0.227	64.6 ± 17	0.235	68 ± 22.7	0.049*	70.1 ± 15.4	0.372	11.5 ± 3.1	0.677
Female (*n* = 152)	66.1 ± 14.2		62.5 ± 16.4		63.6 ± 18.1		68.6 ± 15.3		11.6 ± 2.9	
Physical activity										
Insufficiently active (*n* = 123)	66.1 ± 16.1	0.298	61.8 ± 17.3	0.125	62.9 ± 23.8	0.037*	67.7 ± 14.9	0.116	10.7 ± 3.1	<0.001*
Sufficiently active (*n* = 230)	67.9 ± 14.7		64.7 ± 16.4		67.8 ± 19.1		70.4 ± 15.5		12 ± 2.9	
BMI										
Underweight (*n* = 27)	61 ± 15.1	0.056	60.5 ± 13.2	0.1	64.2 ± 20.9	0.113	70.7 ± 17.3	0.917	10.6 ± 2.7	0.122
Normal weight (*n* = 168)	68.8 ± 14.2		65.8 ± 16.4		67.6 ± 18.6		69.7 ± 15.9		11.3 ± 3.1	
Overweight (*n* = 109)	67.5 ± 16.1		62.9 ± 17.4		67.3 ± 21.3		68.7 ± 15		11.8 ± 3.4	
Obesity (*n* = 49)	64.9 ± 15.9		60 ± 17.8		59.7 ± 26.4		69.8 ± 13.6		12.1 ± 2.1	

*P-value < 0.05.

Physical activity was positively correlated with QoL. Participants engaged in physical activity had higher scores in all domains, with A significant impact on social relations (*p* = 0.037). Sufficiently active participants also had higher diet quality scores (*p* < 0.001), linking exercise to healthier eating habits.

BMI trends indicated higher QoL scores for those with normal weight, though differences were not statistically significant (*p* = 0.056–0.917).

For diet quality, obese participants had the highest diet quality scores (12.1 ± 2.1), while underweight individuals had the lowest (10.6 ± 2.7), but the difference was not significant (*p* = 0.122) (Table 2).

### 3.3 Association of diet quality, physical activity, and demographic variables with quality of life

The relationship between QoL domains and the diet quality (RDQS) scores is displayed in Table 3. All correlation coefficients were weak (*r* < 0.4), but significant positive associations were observed. Physical health correlated with RDQS (*r* = 0.219, *p* < 0.001), suggesting that better diet quality may enhanc the physical well-being of this population. Psychological health also showed a positive correlation (*r* = 0.184, *p* = 0.002), supporting the inclusion of dietary improvements as a part of broader mental health strategies. Social relations had a modest but significant correlation (*r* = 0.109, *p* = 0.041), implying that diet quality influence social interactions. The environment domain also correlated positively (*r* = 0.134, *p* = 0.012), indicating individuals with healthier diets may perceive their surroundings more favorably.

**Table 3:** The relationship between quality of life and diet quality (*n* = 353).

QoL domains	RDQS	
	Correlation	*P*-value
Physical health	0.219**	<0.001
Psychological health	0.184**	<0.002
Social relations	0.109*	0.041
Environment	0.134*	0.012

QoL, quality of life; RDQS, Rapid Diet Quality Screener; Correlation, Pearson correlation coefficient; *P-value < 0.05; **p-value < 0.01.

Table 4 shows that diet quality (RDQS) consistently emerged as the strogest significant predictor across all dimensions of quality of life, including physical (*B* = 1.132, *t* = 4.152, *p* < 0.001), psychological (*B* = 1.002, *t* = 3.341, *p* = 0.001), social relationship (*B* = 0.710, *t* = 1.891, *p* = 0.059, marginally significant), environmental (*B* = 0.690, *t* = 2.465, *p* = 0.014), and overall QoL (*B* = 0.883, *t* = 3.620, *p* < 0.001). In addition, BMI significantly and negatively predicted the psychological (*B* = −0.372, *p* = 0.013), social relationship (*B* = −0.584, *p* = 0.002), and overall QoL dimensions (*B* = −0.296, *p* = 0.015). Gender also showed a significant negative effect on the social (*B* = −6.087, *p* = 0.009) and overall QoL domains (*B* = −3.391, *p* = 0.026), indicating lower scores for females. Other variables, including age and physical activity, did not show significant associations across most QoL domains. All models except for the environmental dimension were statistically significant at *p* < 0.05, with explained variances ranging from 5.9% (physical, psychological, social) to 6.7% (overall QoL), while the environmental model was not significant (*p* = 0.10), explaining only 2.6% of the variance.

**Table 4:** Multiple regression analysis of quality of life domains in relation to diet quality, physical activity levels, and demographic variables (*n* = 353).

Predictor	*B* (Unstd.)	Std. Error	Beta (Std.)	*t*	Sig.	*R*	*R* ^2^	Adjusted *R*^2^	*F* (df)	Sig. (ANOVA)
Physical dimension										
(Constant)	58.900	5.467		10.773	<0.001	0.242	0.059	0.045	4.323 (4, 348)	0.001**
Gender #	−2.808	1.696	−0.091	−1.656	0.099					
Age	0.054	0.142	0.020	0.381	0.703					
BMI	−0.192	0.135	−0.077	−1.425	0.155					
RDQS	1.132	0.273	0.226	4.152	<0.001					
Physical activity	−0.259	1.739	−0.008	−0.149	0.882					
Psychological dimension										
(Constant)	57.497	6.014		9.560	<0.001	0.243	0.059	0.045	4.345 (4, 348)	0.001**
Gender #	−3.439	1.865	−0.102	−1.844	0.066					
Age	0.179	0.156	0.062	1.148	0.252					
BMI	−0.372	0.149	−0.136	−2.505	0.013					
RDQS	1.002	0.300	0.182	3.341	0.001					
Physical activity	0.689	1.913	0.020	0.360	0.719					
Social relationship dimension										
(Constant)	66.692	7.524		8.864	<0.001	0.242	0.058	0.045	4.299 (4, 348)	0.001**
Gender #	−6.087	2.334	−0.144	−2.609	0.009					
Age	0.246	0.195	0.068	1.265	0.207					
BMI	−0.584	0.186	−0.171	−3.141	0.002					
RDQS	0.710	0.375	0.103	1.891	0.059					
Physical activity	2.445	2.393	0.056	1.022	0.308					
Environment dimension										
(Constant)	65.804	5.609		11.731	<0.001	0.162	0.026	0.012	1.865 (4, 348)	0.1
Gender #	−1.229	1.740	−0.040	−0.706	0.480					
Age	−0.147	0.145	−0.055	−1.009	0.313					
BMI	−0.035	0.139	−0.014	−0.249	0.803					
RDQS	0.690	0.280	0.136	2.465	0.014					
Physical activity	1.793	1.784	0.056	1.005	0.316					
Quality of life										
(Constant)	62.223	4.893		12.716	0.000	0.259	0.067	0.054	4.991 (4, 348)	0.001**
Gender #	−3.391	1.518	−0.123	−2.234	0.026					
Age	0.083	0.127	0.035	0.656	0.512					
BMI	−0.296	0.121	−0.132	−2.446	0.015					
RDQS	0.883	0.244	0.196	3.620	0.000					
Physical activity	1.167	1.556	0.041	0.750	0.454					

Dependent variable: dimensions of quality of life, coefficient; *P-value < 0.05, **P-value < 0.01.

# References is male.

## 4 Discussion

To the best of the authors’ knowledge, this is the first Arab study that examines the relationship between quality of life, diet quality, and personal characteristics among healthcare professionals. In Saudi Arabia, healthcare professionals frequently encounter substantial workloads, irregular work schedules, and restricted access to nutritious food options within hospital environments. Additionally, cultural norms may shape gender roles, social expectations, and dietary behaviors, thereby influencing health outcomes (18). A comprehensive understanding of these contextual factors is essential for developing effective interventions specifically tailored to the local healthcare system.

Our findings reveal significant positive correlations between diet quality and various QoL domains. Notably, the social relations domain demonstrated significant differences based on physical activity and gender. Furthermore, participants who regularly engaged in exercise exhibited healthier dietary habits. Diet quality was the strongest and most consistent predictor across all dimensions of quality of life. Lower BMI significantly predicted better psychological, social, and overall QoL, while male gender was a significant predictor of higher social and overall QoL.

Numerous studies have been conducted among healthcare professionals; however, they primarily focus on assessing QoL or lifestyle factors separately. A recent study aimed to evaluate the QoL among 3,170 healthcare workers from 19 Arab countries using the WHOQOL-BREF instrument. It reported that most healthcare workers in the Arab world exhibited unsatisfactory QoL scores across various domains: physical (59.2%), psychological (84.6%), social (73.8%), and environmental (77.7%), except for general QoL, where the score was 38.8%. This underscores the need to focus attention on this group to enhance their productivity and quality of service provision (7). Our study revealed weak but significant positive correlations between healthcare professionals’ diet quality, as measured by the RDQS, and QoL domains, particularly physical (*r* = 0.219) and psychological health (*r* = 0.184), suggesting a diet’s role in overall well-being. Additionally, improved diet quality reliably predicted enhanced quality of life across all domains. These findings align with those of previous studies, suggesting that better diet quality is associated with improved health outcomes, stressing the importance of dietary habits in overall well-being (6, 19). Al Hazmi et al. (18), who investigated dietary habits among healthcare workers in Riyadh, found widespread unhealthy dietary behaviors. A notable percentage of healthcare professionals frequently consume sweets (46.6%) and coffee (66.2%). The study also revealed that Saudis had higher rates of binge eating at home compared to non-Saudis (61.9% vs. 22.5%) (18). Another study on manufacturing employees, not centered on healthcare professionals, has revealed that adherence to a healthy diet is linked to enhanced QoL (20). Additionally, the 2025 systematic review by Godos et al., which incorporated 13 studies of the general population and 15 studies of various patient groups, indicated a significant positive correlation between adherence to the Mediterranean diet and health-related quality of life (HRQoL), particularly in the physical domains (21). Conversely, another study has reported conflicting findings regarding the relationship between healthier dietary habits and quality of life. A cross-sectional study in France assessed the dietary patterns and health-related quality of life (HRQoL) of 308 adult females using three 24-h dietary recalls and a Food Benefit Assessment (FBA) questionnaire. Cluster analysis was employed to identify dietary patterns, while the FBA questionnaire was used to evaluate HRQoL. The study reported no significant differences in QoL based on dietary patterns in a sample of adult females, which they attributed to potential limitations in dietary assessment methods (22).

Nutritional behavior is intricately shaped by various factors, among which nutritional knowledge stands out. Consistent and effective nutrition education is crucial for promoting health and transforming eating habits across all age groups (23). Furthermore, health literacy plays a critical role as a determinant of health-enhancing behaviors (24). Poor health literacy hinders one’s ability to detect and address health problems. A key part of health literacy, nutrition literacy, involves understanding, interpreting, and utilizing basic nutritional information to make informed dietary decisions. The impact of an individual’s knowledge, attitudes, skills, and behaviors related to food and nutrition on their dietary choices is widely recognized. Research has consistently shown that many people struggle to understand information on food labels, and those with limited health literacy or numerical skills often experience adverse health outcomes (25).

In addition, it is believed that physical activity can enhance social well-being, potentially by fostering greater community involvement or promoting social interaction (26); indeed, the findings of our study confirm this, by showing that healthcare professionals who engaged in regular physical activity reported healthier dietary habits and achieved higher scores across all QoL domains, with social relations showing a statistically significant improvement (*p* = 0.037). Although physical activity was not a significant predictor of QoL domains, and no significant differences were observed in the physical health, psychological health, and environmental domains, the trends indicate a general enhancement in QoL for those who are physically active. Also, several studies have revealed that higher physical activity level is related to better QoL (16, 17, 22, 23). Gill et al. (27) demonstrated that engaging in physical activity positively affects all domains of quality of life, extending beyond mere physical improvements to enhance social and emotional well-being, which may primarily motivate community participants. In consistence, Di Bartolomeo and Papa (26) found that regular physical activity significantly improved trust and social connectedness among participants engaged in structured sports activities. Their research highlights the cooperative nature of sports, emphasizing how teamwork, communication, and shared goals foster trust and build stronger social networks. However, this study primarily focused on team sports, which may limit its applicability to other forms of physical activity, such as yoga or walking, that lack structured group dynamics. Additionally, individual differences such as personality traits or preexisting social skills were not considered, which may moderate the social benefits derived from physical activity (26).

Further evidence supporting the beneficial effects of physical activity on health care professionals’ QoL can be seen in a study by Grimani et al. (1), who demonstrated that workplace exercise programs enhanced not only physical health but also mental well-being and work performance among health care staff (3). However, some studies have reported inconsistent results. For example, Sharma et al. (4) found that while physical activity improved physical health indicators in healthcare professionals, its impact on psychological well-being was limited due to ongoing work-related stress factors. Similarly, Zubair et al. (28) stated no significant correlation between physical activity levels and QoL among surgical residents, attributing this lack of impact to extended working hours, irregular schedules, and burnout, which could potentially overshadow the benefits of exercising. These findings suggest that, although physical activity is generally advantageous, its positive effects may be diminished by occupation-specific factors in healthcare environments.

Another interesting finding of this study is the variability of the scores by gender. Being male significantly predicted better the social and overall quality of life. Male participants demonstrated marginally higher scores than female participants across all QoL domains (physical health, psychological health, social relations, and environment), with a statistically significant difference observed in social relations. This suggesting that gender influences the social aspects of QoL among medical staff. This finding is consistent with those of other studies conducted in Greece, Italy, the USA, and Oman (29–31). Tountas et al. (29) linked gender disparities in health-related quality of life to the lower social status of female in a male-dominated society, particularly in Greece, a Mediterranean country where traditional norms further diminish female’s standing. These disparities may also reflect professional differences, as most males in this study were medical doctors, whereas women primarily held nursing or auxiliary positions (29). As previously mentioned, our study found that males generally scored higher than females in all QoL domains. However, the only statistically significant difference occurred in social relations. The lack of significant results in other areas may reflect substantial ongoing efforts to empower females, aligning with one of the objectives of Saudi Vision 2030 (32).

In our study, dietary quality did not show significant differences between the genders, indicating that both maintain comparable dietary practices (3, 16). However, previous research has highlighted poor dietary habits among healthcare workers in Riyadh, with females consuming more fruits and vegetables than males (47.1%, 18.6%, *p* < 0.000) (18).

Although previous research has suggested that maintaining a normal BMI is linked to improved QoL outcomes (29, 30), our findings revealed that lower BMI significantly predicted better psychological, social relationship, and overall QoL dimensions. Additionally, our study observed a trend where participants with normal weight had the highest mean scores across QoL domains. In contrast, those who were underweight or obese had lower scores. However, no statistically significant differences were observed in the mean scores across various BMI categories and QoL domains. This aligns with the existing literature that does not establish a definitive correlation between BMI and QoL (33).

Finally, numerous studies have highlighted that the well-being of healthcare practitioners significantly impacts the quality and safety of patient care (34, 35). Enhanced well-being among healthcare professionals is associated with improved care delivery (34). Additionally, the well-being and quality of work environment have been identified as critical factors affecting patient care outcomes (31). This emphasizes the importance for hospital management and policymakers to focus on improving work-related factors, thereby boosting healthcare professionals’ well-being and, consequently, elevating the standard of patient care.

This study has several strengths marks a significant contribution to the literature on diet quality and QoL among this population. First, diet, QoL, and physical activity were evaluated using validated instruments (12–17). Second, we used structured interviews to collect data (considered more reliable than self-administered questionnaires for assessing behaviors). Third, both sexes were included in the present study. However, there are some limitations that should be considered. First, it employed a cross-sectional design that precludes causal inferences. Finally, weight and height were self-reported, which could have created recall bias due to over- or under-reporting, potentially affecting the accuracy of our results.

## 5 Conclusions and future implications

Our findings highlight the interrelation between physical activity, diet quality, and QoL, suggesting that interventions aimed at improving dietary habit and lifestyle choices among medical staff could enhance individual health outcomes, improve social and psychological well-being, and ultimately enhancing the quality of patient care. With growing global attention on the well-being of healthcare workers, these findings have broad international relevance, reinforcing the role of dietary interventions in enhanceing QoL in healthcare settings. It is also important to consider potential confounding variables not fully accounted for in this analysis, such as occupational stress, sleep patterns, specific job roles, and work hours. These factors may influence dietary habits and QoL and should be explored using multivariate approaches in future studies.

Future research should investigate these relationships more comprehensively through longitudinal studies to establish stronger causal links. Such studies would provide more comprehensive insights, forming the basis for evidence-based policies aimed at improving healthcare professionals’ QoL in Saudi Arabia and beyond.

Incorporating nutrition education into public health initiatives and promoting awareness of nutrition and sustainability are essential for supporting informed dietary choices. Implementing evidence-based practices and policies supported by collaborative efforts is crucial for improving the diet quality and overall well-being of healthcare professionals. Integrating physical activity facilities within a medical institution can encourage staff to engage in physical activity during breaks and working hours. This enhancement can positively impact the emotional, social, and potentially spiritual aspects of life, contributing to a fulfilling experience. Ultimately, this could lead to improved QoL for healthcare professionals.

Finally, this study highlights the importance of implementing policies and initiatives to enhance healthcare professionals’ well-being and quality of work life. Strengthening these areas can contribute to a healthier workforce, ultimately enhancing patient care and the overall effectiveness of healthcare systems.

## Data Availability

The datasets generated during and/or analyzed during this study are not publicly available due to data protection requirements. Requests to access the datasets should be directed to WA, Walfawaz@ksu.edu.sa.
